# Alterations of Heartbeat Evoked Magnetic Fields Induced by Sounds of Disgust

**DOI:** 10.3389/fpsyt.2020.00683

**Published:** 2020-07-22

**Authors:** Yutaka Kato, Yuichi Takei, Satoshi Umeda, Masaru Mimura, Masato Fukuda

**Affiliations:** ^1^ Tsutsuji Mental Hospital, Gunma, Japan; ^2^ Department of Psychiatry and Neuroscience, Gunma University Graduate School of Medicine, Maebashi, Japan; ^3^ Department of Psychology, Keio University, Tokyo, Japan; ^4^ Department of Neuropsychiatry, Keio University School of Medicine, Tokyo, Japan

**Keywords:** MEG (magnetoencephalography), insular cortices, emotion, heartbeat evoked magnetic fields, cortical connectivity, oscillation

## Abstract

The majority of the models of emotional processing attribute subjective emotional feelings to physiological changes in the internal milieu, which are sensed by the interoceptive system. These physiological reactions evoked by emotional phenomena occur *via* the autonomic nervous system, and give rise to alterations in body-mind interactions that are characterized by heartbeat evoked magnetic fields (HEFs) involving brain regions associated with emotional perception. The current study used magnetoencephalography (MEG) to examine regional cortical activity and connectivity changes in HEFs provoked by the emotion of disgust. MEG results from 39 healthy subjects (22 female) revealed that passively listening to sounds of disgust elicited right insular cortical activity and enhancement of cortical connectivity between the right anterior ventral insular cortex and left ventromedial prefrontal cortex, demonstrated by phase lag indexes in the beta frequency range. Furthermore, inter-trial coherence significantly increased at 19 Hz and 23 Hz, and decreased at 14 Hz, which highlights the involvement of low beta oscillations in emotional processing. As these results were based on spontaneously triggered bioelectrical signals, more indigenous and induced signals were extracted with a block designed experiment. The insular cortices play an important role in emotional regulation and perception as the main cortical target for signals with interoceptive information, providing direct substrates of emotional feelings. The current results provide a novel insight into frequency properties of emotional processing, and suggest that emotional arousal evoked by listening to sounds of disgust partially impact the autonomic nervous system, altering HEFs *via* connectivity changes in the right anterior ventral insular cortex and left ventromedial prefrontal cortex.

## Introduction

The majority of the models of emotional processing attribute subjective emotional feelings to physiological changes in the internal milieu, including the skeletomuscular, neuroendocrine, and autonomic nervous systems (1–3). These changes are often triggered by external events as well as the homeostatic changes of inner bodily status, and can affect body-mind interactions (4–6). Indeed, affective experiences initially give rise to specific physiological changes in the body, which are bidirectionally associated with the perception of one's emotions (7, 8). Central representations of the bodily responses caused by external emotive stimuli are perceived as emotions; i.e., automatically generated bodily responses *via* the autonomic nervous system can result in subjective emotional feelings (9). Derived from this theory is the notion that the relative ability to perceive internal body status influences measures of subjective affective experience, which highlights the fundamental importance of the body in emotional phenomena.

The ability to self-monitor one's own internal body status is a key feature of the body-mind interaction in emotional awareness. Interoception, which is the sensitivity to visceral sensations (10), refers to the current homeostatic conditions of the entire body (11) and the ability of this information to reach conscious awareness (12). Interoception derives not only from musculoskeletal, circulating, and humoral signals within the body, but also from afferent information *via* autonomic nervous reflexes and higher levels of autonomic regulation originating from visceral organs and vascular systems (13). Two distinct neural pathways convey interoceptive information. The lamina I spinothalamocortical pathway carries information regarding the internal milieu, such as muscle contractions in vessel walls, peripheral blood flow, temperature, pain, tissue injury, and levels of O2 and CO2, while the vagal nerve carries visceral signals regarding the cardiovascular, respiratory, gastrointestinal, and genito-urinary systems (14). The crosstalk between these two pathways within the projections of the brainstem to the insular and somatosensory cortices facilitates the organization of topographical maps of bodily status within structures (3). Namely, interoceptive information originates from multiple organs *via* corresponding conduits and is gathered and successively processed in the structures of the brain, providing topographically organized somatic maps. Among these, the sensitivity to one's own heartbeat is closely associated with interoceptive awareness (14, 15) and the intensity of experienced emotions (16–18). The accuracy of heartbeat counts has been found to be positively correlated with the sensitivity of emotional traits, such as tendencies for general anxiety (19, 20).

To investigate the cortical activation related to interoception, both heartbeat evoked potentials (HEPs) and heartbeat evoked magnetic fields (HEFs), which are measured using electroencephalography (EEG) and magnetoencephalography (MEG), respectively, serve as useful indexes of interoceptive processing, and reflect the sensitivity to ones' own internal body states (21–23). HEPs/HEFs are computed by averaging the neural signals with time-locked triggers of individual R peaks of electrocardiography (ECG), which emerge after 200 to 300 ms and are mainly derived from fronto-central regions of the brain (24–29). Thus far, HEPs/HEFs have been associated with cardiovascular arousal (30), heartbeat awareness (31–33), visual awareness (34), self-consciousness (35, 36), self-face recognition (37), pain perception (38), empathy (39), sleep states (40), and psychiatric disorders, including depression (41), depersonalization (42), and borderline personality disorder (43).

Aside from this accumulating evidence, the precise mechanisms of HEPs/HEFs on emotional experiences are still unclear. As most studies have used EEG to measure the effects on interoceptive awareness, poor spatial resolution has obscured elucidation of these physiological features. We hypothesized that the subjective experience of emotions would elicit the peripheral physiological responses initially, then affect the emotion related cortical activities. Particularly, we anticipated that disgust feeling of emotion will impact either myocardial or gastric functions, thus give rise to the alteration of HEFs reflecting these changes of bodily status. To assess our hypothesis that spontaneous heartbeat movements evoke cortical activities and connectivity changes related to emotional processing, we conducted MEG, which has a high temporal and spatial resolution. In this way, we investigated alterations in HEFs that are mediated by disgusting experiences to clarify the precise cortical activity, connectivity, and frequency properties to better understand brain-mind interactions.

## Materials and Methods

### Subjects

Thirty-nine healthy volunteers participated in the experiment (22 female, mean age: 39.3 years ± 8.55 years, range: 20–58 years). One additional participant was excluded because of technical artifacts during the MEG recording. All participants had normal or corrected-to-normal vision and no hearing difficulties, and all were right-handed according to the Edinburgh Handedness Scale (44). No participants reported any history of neurological or major psychiatric conditions, and no participants were on medications, including mood stabilizers, antidepressants, antipsychotics, anxiolytics, or hypnotics. Before the experiment, all subjects were fully informed about the MEG recording, and they all provided written informed consent. The experimental procedures were approved by the Ethics Committee of Gunma University Graduate School of Medicine in concordance with the Code of Ethics of the World Medical Association (Declaration of Helsinki).

### Stimuli

**Figure 1:**
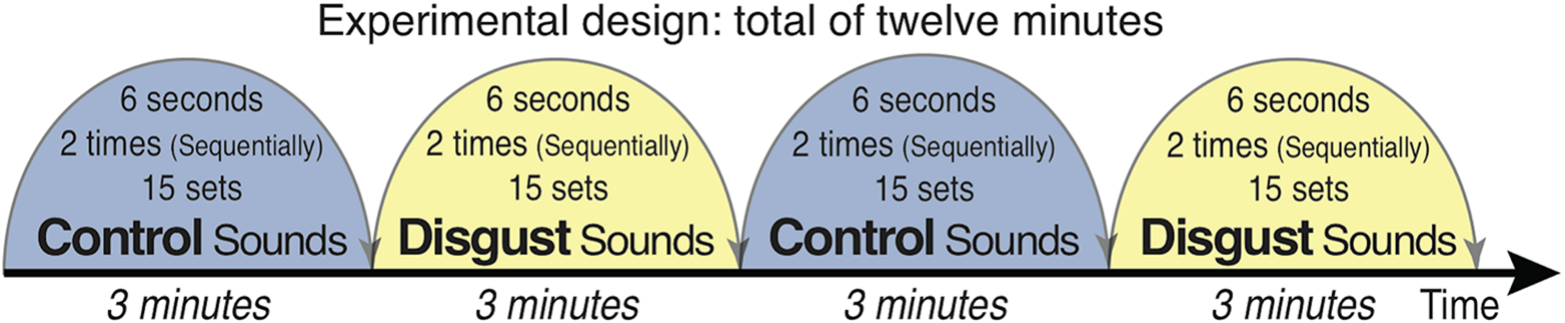
Schema of the experimental design. Auditory stimuli were presented in a shielded room *via* earphones bilaterally while participants fixated on a cross. Ten types of disgust/control sounds were presented from the International Affective Digital Sounds (including human vomiting/cow moos, human fighting with a stomach being hit/digging a hole with a shovel, and child abuse/cheers in a baseball stadium). Each of the 15 stimuli (6 s) was presented twice (12 s), with 3 min of stimulus presentation per block. The blocks were alternatively presented, and the experiment lasted for a total of 12 min.

Subjects passively listened to auditory sounds of disgust and control sounds selected from the International Affective Digital Sounds (IADS-2) (45) with their eyes open and focused on a fixation point projected on a screen during the presentations. The experiment consisted of four blocks of 3 min each, in which two different types of sound sets were alternatively presented; the entire experiment therefore took 12 min in total (**Figure 1**). The auditory stimuli of disgust consisted of a 6-second stereo audio recording of scenic events that effectively provoked the affective scenery (successively presented twice: 12 s) with 15 sets of sounds (one session forming blocks with 3 min of stimuli each), which were randomly selected from 10 different sound sets. The 10 sounds of disgust were as follows: (1) Male Cough (IADS-2 sound No. 241; a man having a coughing fit), (2) Female Cough (No. 242; a woman having a coughing fit), (3) Hiccups (No. 245; a man with sustained hiccups), (4) Vomiting (No. 255; a man vomiting), (5) Screaming (No. 275; a man screaming), (6) FemScream2 (No. 276; a female screaming for help), (7) Child Abuse (No. 278; a baby screaming after the sound of being hit by a man using abusive language), (8) Attack3 (No. 281; sounds of domestic violence indicating that a woman is being hit), (9) Fight3 (No. 283; a fight between two men as indicated by sounds of abdominal hitting), and (10) Belch (No. 702; a man belching).

Control sounds were selected with the same sound set structure as that of the sounds of disgust. The 10 control sounds were as follows: (1) Baby (IADS-2 sound file No. 110; a baby laughing), (2) Music Box (No. 111; a baby laughing with music box sounds), (3) Kids1 (No. 112; voices of pleasure of children in the park), (4) Cows (No. 113; a cow mooing similar to the sound of vomiting), (5) Pig (No. 130; barks of pigs similar to belching sounds), (6) Glowl2 (No. 133; the bark of an elephant), (7) Brook (No. 172; the sound of water flowing), (8) Kids2 (No. 224; the sounds of playing children in the park), (9) Baseball (No. 353; cheering sounds in a baseball stadium followed by the sound of something being hit, similar to the sound of child abuse), and (10) Shovel (No. 382; the sound of digging a hole with a shovel, similar to the sound of abdominal hitting).

After the experiment, subjects were requested to rate the strengths of emotions they had felt (maximum 100; minimum 0) during the disgust and control blocks, respectively, regarding six elements of emotions – fear, sadness, anger, disgust, happiness, and love (46). Statistical analysis was performed by scaling the independent components of emotions according to the disgust/control conditions.

### MEG Recording

MEG was recorded in a magnetically shielded room (JFE Mechanical Co., Tokyo, Japan) with a 306-channel whole-head system (Elekta Neuromag, Helsinki, Finland). The system had 102 sensor-triplets, each of which contained a magnetometer, a longitudinal gradiometer, and a latitudinal gradiometer. Silver cup electrodes were located at six sites of the body to measure individual ECGs and exclude noise from the ECG and EOG. The locations of three fiduciary points (nasion and left and right auricular points) defining the head frame coordinate system, a set of head surface points, and the locations of the four head position indicator coils were digitized using an Isotrak three-dimensional digitizer (Polhemus™, Colchester, VT, USA). MEG and EOG signals were simultaneously recorded during stimuli presentation at a sampling rate of 1,000 Hz. All off-line analyses were based on the saved continuous raw data. Sleepiness scores at the time of the MEG examination were assessed using the Stanford Sleepiness Scale (47) before and after each task.

### Preprocessing and Epoching

Initially, all off-line analyses were based on the saved continuous raw data. For noise suppression and motion correction, the data were spatially filtered using the signal space separation method (48, 49) with Elekta Neuromag Maxfilter software, which suppresses noise generated by sources outside the brain. A notch filter was applied to eliminate noise from the power line (50 Hz) and its harmonics (100 Hz and 150 Hz). We eliminated eye movement- and body movements- related artifacts from the raw data using independent component analysis (ICA). The ICA was applied to the MEG sensor signals, and the eye movement and body movement signals were isolated based on visual inspection by two expert researchers. MEG data were then divided into two epochs, disgust and control conditions, with simultaneously recorded ECG signals, which resulted in two different data sets of 6 min each per condition.

### Data Analysis

HEFs were calculated by MEG signals locked to the R peaks of the ECG separately for the two conditions. We detected R peaks using the “find_ecg_events” function in MNE-python. We acquired epochs of 800 ms, which included a pre-R peak baseline of 200 ms, triggered with ECG R peaks at a latency of 0 ms. According to the rate of the subjects' heartbeat, an averaging of 410 to 570 times provided two different data sets corresponding to the conditions (disgust or control). Subtracted waveforms derived from the subtraction of the control condition from the disgust condition were calculated for every subject. Epochs were rejected if the peak-to-peak amplitude during the target epoching period exceeded 400 fT and 4,000 fT/cm in any of the magnetometer and gradiometer channels, respectively. Using the data of individual ECG, the standard deviation of the averaged normal-to-normal (NN) beat intervals, with both standard deviation of all NN intervals (SDNN) representing sympathetic function, and root mean square of successive differences of successive NN intervals (RMSSD) representing parasympathetic functions, were calculated.

To obtain anatomical segmentation and brain surface images, we first applied the FreeSurfer reconstruction process to each participant's MRI data. Cortical reconstructions and parcellations for each subject were generated from a T1-weighted MRI (50, 51). After correcting the topological defects, cortical surfaces were triangulated using dense meshes with ~130,000 vertices in each hemisphere.

The dense triangulation of the folded cortical surface provided by FreeSurfer was decimated to a grid of 10,242 dipoles per hemisphere, corresponding to a spacing of approximately 3 mm between adjacent source locations. To compute the forward solution, the boundary-element method with a single compartment bounded by the inner surface of the skull was used (52). The watershed algorithm in FreeSurfer was used to generate the inner skull surface triangulations from the anatomical MRI images of each participant. The source current distribution was estimated using the minimum norm estimates (MNE) suite and MNE python, which are widely used distributed source models for MEG analysis (http://www.martinos.org/mne/) (53). We employed the boundary-element method, with a single compartment bound by the inner surface of the skull for the forward head modeling (52). The source current distribution was estimated using the noise-normalized dynamic statistical parameter mapping (dSPM) method (54, 55). The noise covariance matrix was computed from the empty room acquisition data. To reduce the bias of the estimated source locations toward the superficial currents, we incorporated a depth weighting parameter (depth = 0.8) by adjusting the source covariance matrix to evaluate the current (56).

Next, we imported commonly used cortex segmentation from the FreeSurfer software. We used the Desikan-Killiany Atlas (aparc) for cortical parcellation (57, 58). We found prominent activity in the insular cortices, and so parcellated this structure into three sub-regions (anterior ventral, anterior dorsal, and posterior insular cortices) according to a previous study (59). We acquired 72 source waveforms for each participant.

Induced power (Power) and inter-trial coherence (ITC) estimation were acquired using “source_induced_power” function in mne-python (60). Inter-trial coherence (ITC) was indexed by summation of phase angles of all epochs demonstrating as the following formula:

$$ITC\left(f,t\right)=\frac{1}{n}\sum_{k=1}^{n}\frac{F_{k}\left(f,t\right)}{\left|F_{k}\left(f,t\right)\right|}$$

The *F_k_*(*f,t*) denotes the spectral estimate of trial k at frequency f and time, n is the number of trials, || represents the complex norm. The ITC measure takes values between 0 and 1. The Morlet wavelet transform was set to single epochs in time courses in the 8–24 Hz frequency range for a duration of -200 to 600 ms under the arousal and control conditions. Data for Power and ITC in each condition were then normalized against the baseline power between the latencies of 200 ms and 400 ms obtained from the right anterior ventral insular cortices in the control condition.

The phase lag index (PLI) was calculated based on the time series of six insula seed sub-regions between 200 ms and 500 ms at 8–13 Hz for the alpha band and at 14–24 Hz for the beta band (61). Connectivity strength at each region of interest (ROI) was estimated using Fisher's Z-transformed PLI values between that ROI and all other ROIs.

Statistical comparisons were made using Scipy (62) by using statistical parametric maps obtained from MEG data to calculate the source-evoked time courses and ITC across all subjects. Independent t-tests were applied to the source-evoked time courses to compare the two conditions (disgust/control) between -100 and 500 ms. We first normalized the ITC of all insular sub-regions by calculating Z-values using the mean and standard deviations of the ITC in the right anterior ventral insular sub-regions between the 200 and 600 ms latency window and 8 and 24 Hz frequency range (**Figure 3** lower panel). Then, we compared the normalized ITC between the two conditions (disgust/control) using independent t-tests. We considered statistical significance to be indicated by p-values under 1% (enclosed area with white line in **Figure 3**, lower panel; distribution of significance). Next, we compared the averaged values of distribution of significance in each frequency band using independent t-tests. In addition, we compared the PLI of the right anterior ventral insular cortices as the seed region using Mann-Whitney U tests. We masked p-values under 5%, and converted p-values to z-values, as seen in **Figure 5**.

### Data and Code Availability Statement

The raw data supporting the conclusions of this article will be made available by the authors, without undue reservation.

## Results

### Subjective Emotions Evoked by Stimuli

Subjects independently rated their emotional experiences during control/disgust blocks from 0 (minimum) to 100 (maximum) regarding the six elements of emotion (i.e., fear, sadness, anger, disgust, happiness, and love). During disgust conditions, the strengths of emotions such as disgust (average +81.7 ± 25.3), fear (average +40.8 ± 29.3), sadness (average +37.0 ± 35.4), and anger (average +32.2 ± 30.5) were higher. On the contrary, the strengths of happiness (average -43.0 ± 33.5) and love (average -39.7 ± 31.7) were lower. The most dominant emotion evoked by stimuli sounds was disgust, followed by the decreased emotion of happiness.

### Heart Rate Variability

In order to estimate the time-domain measures of heart rate variability, we calculated heart rates, and normal-to-normal beat (NN) intervals to obtain standard deviations for all NN intervals (SDNN), which represent sympathetic function, and the root mean square of successive differences of successive NN intervals (RMSSD), representing parasympathetic function. No significant differences between conditions were found in heart rate (disgust = 66.63 [SD = 8.92], control = 66.67 [SD = 8.95]; p = 0.79), SDNN (disgust = 42.64 [SD = 28.35]; control = 43.87 [SD = 29.47]; p = 0.42), or RMSSD (disgust = 35.06 [SD = 24.93]; control = 35.64 [SD = 23.00]; p = 0.37).

### Source Current Distribution

**Figure 2:**
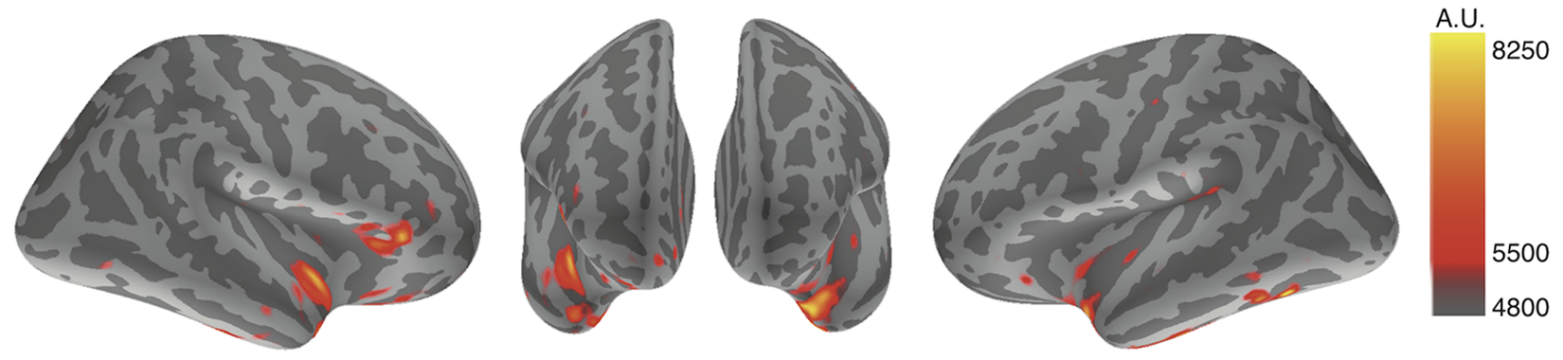
Source current estimation. The source current estimation demonstrated activity in bilateral insular cortices and the right inferior frontal gyrus at the latency of 291.8 ms after the R peak of ECG in the disgust condition. This is illustrated by the source current distributions of disgust minus control conditions of the right, anterior, and left surfaces. The color bar indicates the arbitrary unit (A.U.) defined by mne analyze.

Using MNE analysis (53), cortical strained minimum norm source currents were estimated according to cortical activities during the designated time points throughout the whole averaged latencies. All subjects exhibited cortical activity in both conditions; however, the most prominent findings were derived from the subtracted distributions between the disgust and control conditions, whereby subtracted activity was mainly elicited in the bilateral insular cortices and the right inferior frontal gyrus. Using averaged data across all subjects, we observed increased activity in the bilateral insular cortices and the right inferior frontal gyrus between 270 ms and 300 ms in the disgust condition relative to the control condition. **Figure 2** depicts the subtracted distributions of source current estimation across all subjects at the latencies of 291.8 ms of the right, anterior, and left surfaces order from right to left; the resulting images indicate that the bilateral insular cortices (right dominantly) and the right inferior frontal gyrus were simultaneously activated.

### Source-Evoked Time Courses and Inter-Trial Coherences of Insular Cortical Sub-Regions

**Figure 3:**
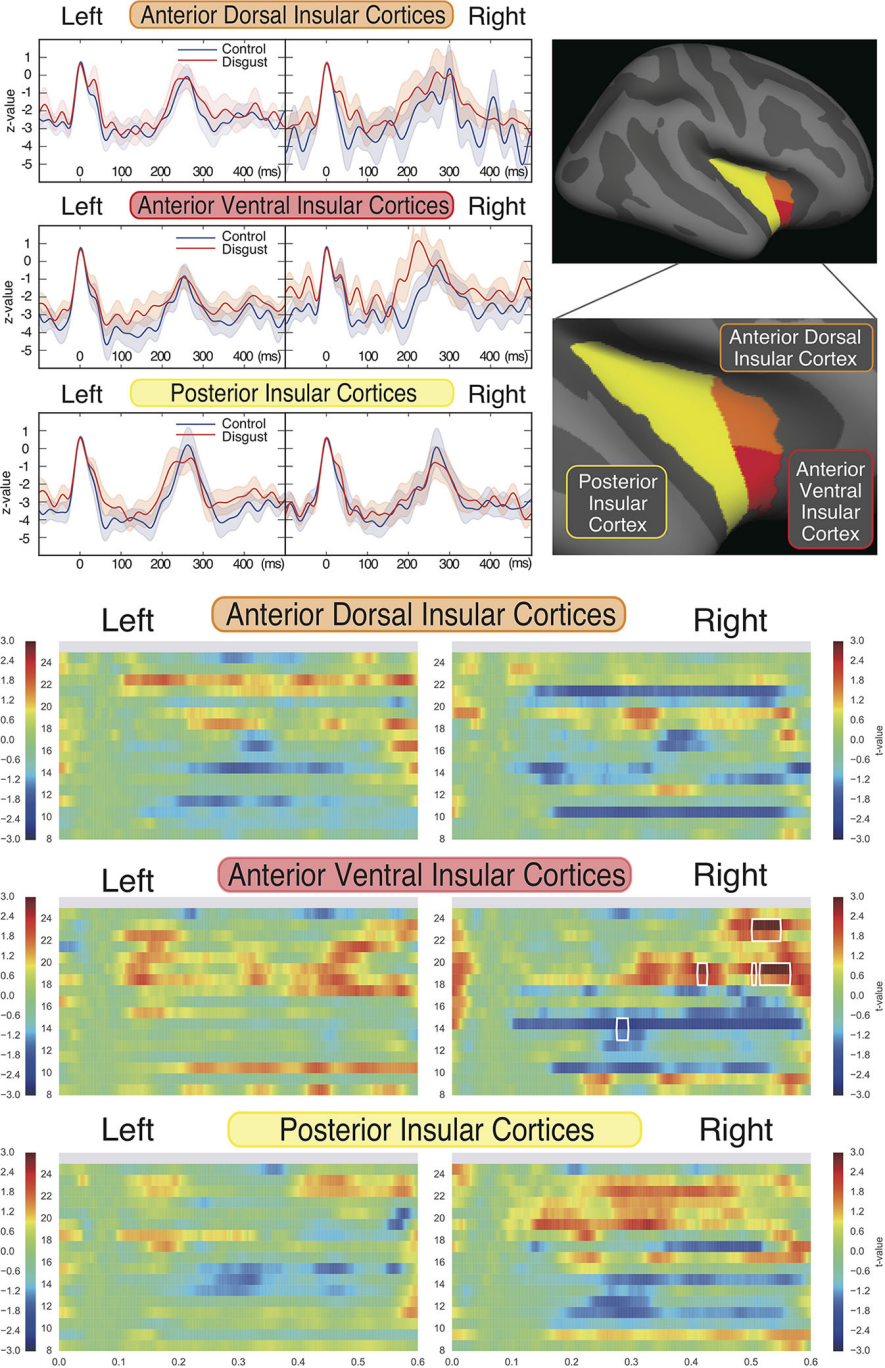
Source strengths of insular sub-regions represented by source-evoked time courses and inter-trial coherences. Insular cortices were divided into three sub-regions, as the anterior ventral, anterior dorsal, and posterior regions of each hemisphere (upper right panel). The source-evoked time courses (upper left panel) were estimated from three sub-regions of the insular cortices of both hemispheres. The X-axis shows the latencies from -100 ms to 500 ms, and the Y-axis shows the Z-value of evoked source activities. The red and blue lines represent the disgust and control conditions, respectively. Z-values were similar at five insular sub-regions; however, the right anterior ventral sub-region demonstrated a trend towards amplification of z-value between 160 ms and 260 ms in the disgust condition. Although no significant differences were found between conditions of all sub-regions, all insular sub-regions exhibited peak amplitudes in both conditions between 250 ms and 300 ms after ECG R peaks. The comparison of inter-trial coherences (ITCs) of the sub-regions (lower panel) between conditions revealed a significant increase of z-value at the frequencies of 19 and 23 Hz and a significant decrease at 14 Hz, only at the right anterior ventral insular sub-regions (highlighted by surrounding rectangles drawn by white line; distributions of significances). The X-axis shows the latencies from 0 ms to 600 ms, the Y-axis shows frequencies from 8 Hz to 24 Hz, and the color bar indicates t-values (+ 3 to - 3) between conditions of each frequency and latency of the responses calculated by t-tests applied for between-condition comparisons (disgust vs. control).

To characterize the insular cortical activity, we evaluated the source-evoked time courses from the insular cortices, which were divided into three sub-regions according to previous studies (59, 63, 64); these sub-regions were the anterior dorsal, anterior ventral, and posterior insular cortices. The mean coordinates of the insular sub-regions were as follows: left anterior dorsal insula (x = -38, y = 6, z = 2), right anterior dorsal insula (x = 35, y = 7, z = 3), left anterior ventral insula (x = -33, y = 13, z = -7), right anterior ventral insula (x = 32, y = 10, z = -6), left posterior insula (x = -38, y = -6, z = 5), and right posterior insula (x = 35, y = -11, z = 6). **Figure 3** (upper right panel) depicts the schema of three insular cortical sub-regions in the right hemisphere. **Figure 3** (upper left panel) depicts the source-evoked time courses of both hemispheres. Although five sub-regions of the insular cortices demonstrated similar activation during whole time course at all frequency ranges, there was a non-significant amplification of activity in the right anterior ventral insular cortices between 160 ms and 260 ms in the disgust conditions (p = 0.274). All six sub-regions of the insular cortices had peak amplitudes at around 250 ms between the 200 ms and 300 ms latency, which suggests that the bilateral insular cortices were similarly activated after the ECG R peaks in both conditions. No significant between-condition differences were found in any of the six sub-regions.

**Figure 4:**
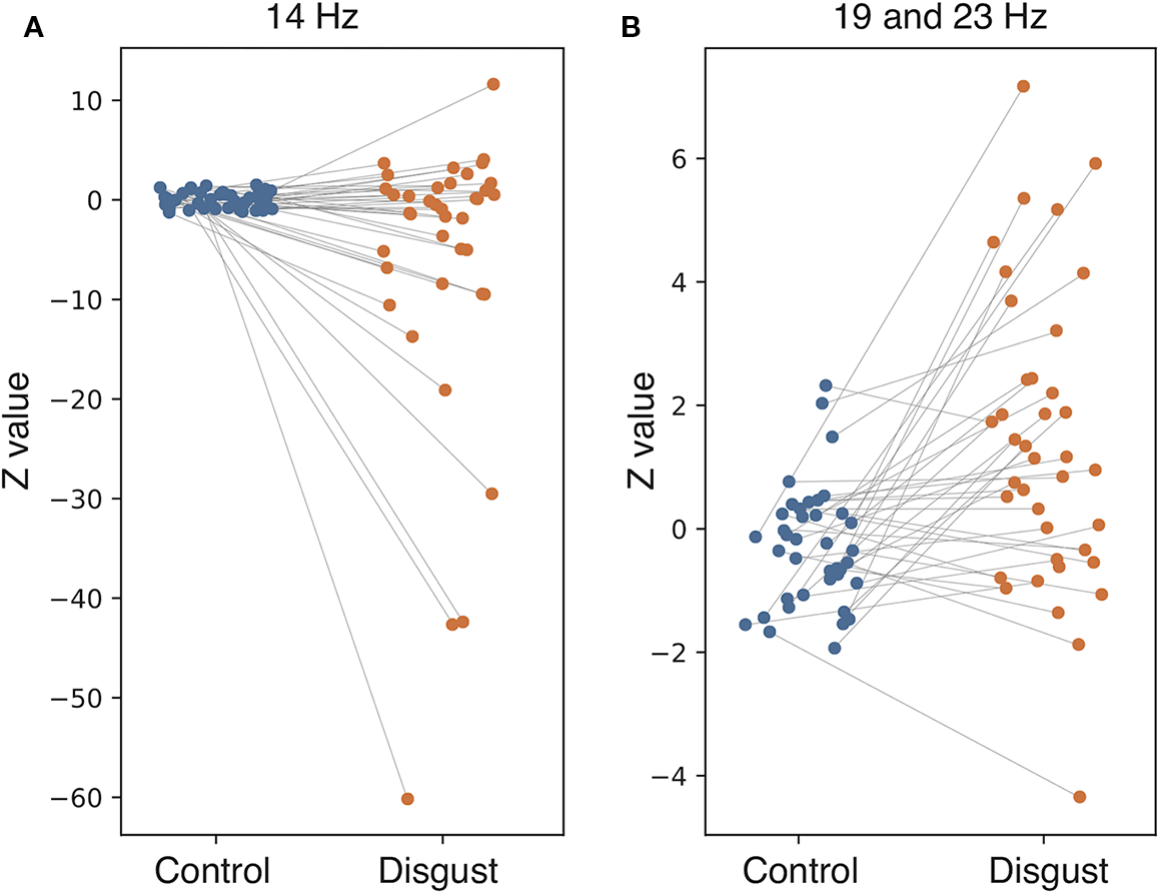
Individual averaged inter-trial coherence (ITC) of distributions of significances. Individual averaged ITCs were plotted for subjects in both conditions (blue plot: control condition; orange plot: disgust condition; line: individual changes between conditions). The **(A)** represents the distributions at 14 Hz, and the **(B)** shows distributions at 19 and 23 Hz. Each plot indicates the Z-value of individual averaged ITC in both conditions corresponding to the individual changes.

To investigate the frequency properties of these responses, we calculated the inter-trial coherence (ITC) from the regional source power analysis in 12 different distributions (six regions by two conditions) according to the insular sub-regions. Statistical comparisons of each ITC between the two conditions (disgust/control) were made at the frequency range of 8 to 24 Hz in the 0 ms to 600 ms latency window by normalizing the baseline power between the 200 ms and 600 ms latency obtained from the right anterior ventral insular cortices of the control conditions, shown by the t-values between conditions in **Figure 3** (lower panel). Significant differences between the control and disgust conditions were found at five distributions (distributions of significances) in only right anterior ventral insular sub-region responses; there were significant increases in four distributions at the frequencies of 19 Hz at around 400 ms, 500 ms, and 550 ms, and 23 Hz at around 525 ms (t-value = 4.064, p = 0.00012; disgust mean = 1.380 [SD = 2.38]/control mean = -0.294 [SD = 0.97]). There was a significant decrease in one distribution at 14 Hz at around 300 ms (t-value = -2.676, p = 0.00912; disgust mean = -6.131 [SD = 14.43]/control mean = 0.0632 [SD = 0.83]), highlighted by surrounding white rectangles in **Figure 3** at the panel of right anterior ventral insular cortices. There were no significant differences between conditions in the other insular sub-regions at any frequency range or latency. The individual averaged ITCs of two low beta bands (14 Hz; 19 and 23 Hz) in five significantly different distributions (distributions of significances) of each condition are shown in **Figure 4** for a total of 39 subjects. The left panel represents the averaged ITC of the frequency range of 14 Hz individually (disgust mean = -6.13 [SD = 14.4]/control mean = 0.063 [SD = 0.83]), whereas the right panel shows a frequency range of 19 and 23 Hz (disgust mean = 1.38 [SD = 2.38]/control mean = -0.29 [SD = 0.97]); in both frequencies, there were significant differences between the conditions according to the statistical significances of the averaged ITC. These individual averaged ITC were not correlated to z-scores of visual analog scales statistically of each emotion, that subjects felt during stimuli presentations.

### Phase Lag Index

**Figure 5:**
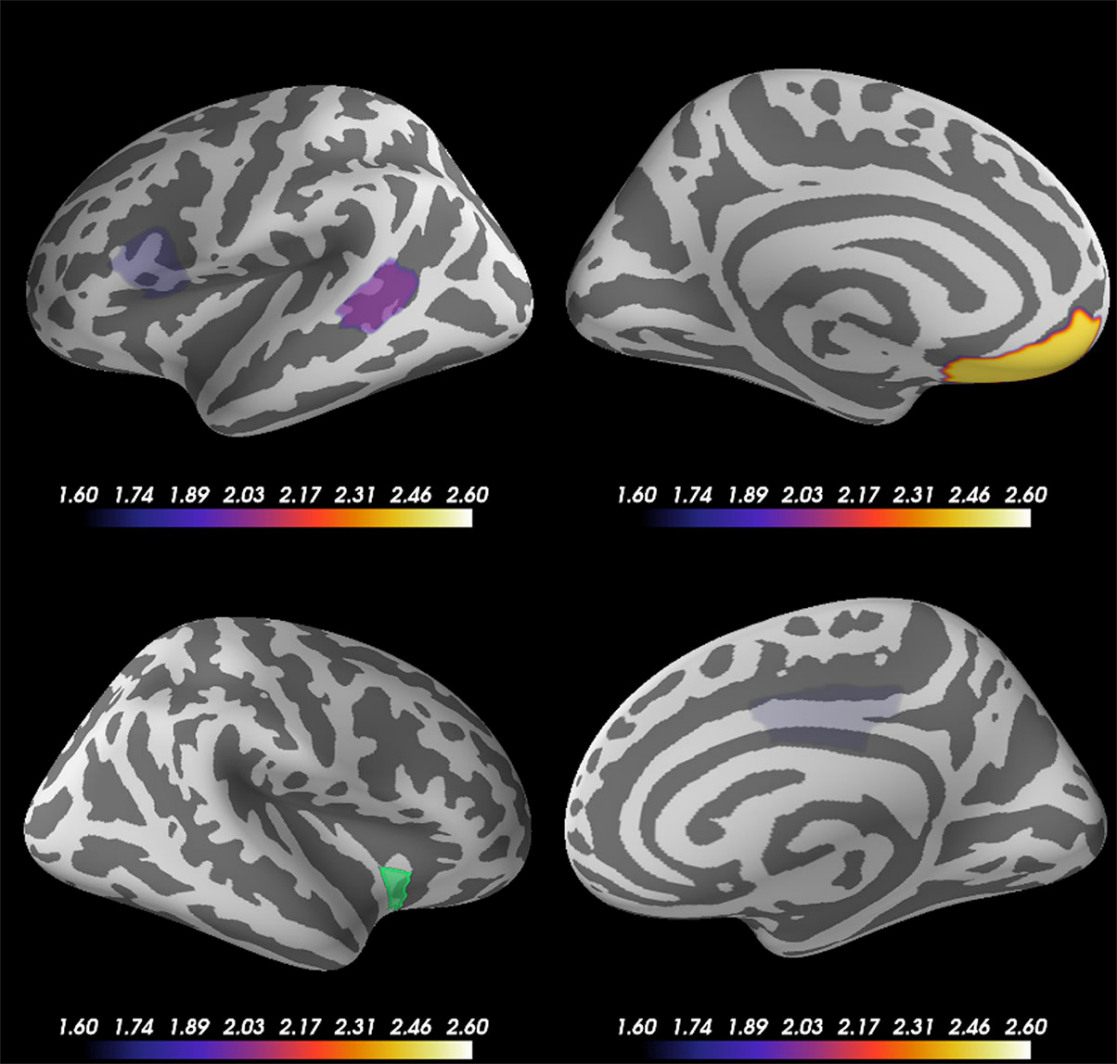
Cortical regions showing significant changes in the phase lag index (PLI) between the right anterior ventral insular cortices. Statistical comparison between the two conditions (disgust/control) of the right anterior ventral sub-region of insular cortices and other brain regions (68 vertexes) using phase lag indexes (PLI) demonstrated significant increases between the right anterior ventral region of the insular cortices (green) and left ventromedial prefrontal cortex (VMPFC) at the beta frequency range of 14 Hz to 24 Hz. The other brain regions that exhibited significant changes of PLI between the right anterior ventral insular cortices were the left pars opercularis, right posterior cingulate, and left bank of the superior temporal sulcus. The color bar indicates the z-value determined using the Mann-Whitney U test of PLI between the right anterior ventral insular cortex and other cortical regions by conditions.

Based on the source-evoked time courses and ITC results, which indicated the importance of the beta frequency range, particularly at the right anterior ventral insular sub-region, we calculated the phase lag indexes (PLIs) between right anterior ventral insular cortical sub-regions and other cortical regions of the 68 vertexes across the whole brain at beta frequency bands (14–24 Hz) across all subjects. To clarify the significant correlation of PLI to other brain regions, we excluded PLIs with p-values exceeding 0.05 according to the Mann-Whitney U test. **Figure 5** shows the subtracted PLI between the disgust and control conditions at the beta frequency range between the right anterior ventral insular cortices and other brain regions. Significant connectivity changes were found at the contralateral (left) ventromedial prefrontal cortex (VMPFC) in concordance with the right anterior ventral region of the insular cortices at the beta frequency range when comparing the two conditions using the PLI result (Mann-Whitney U = 284.0, p = 0.0072, disgust mean = 0.0656 [SD = 0.0441]/control mean = 0.0450 [SD = 0.0323]). Significant increases in PLI compared to the anterior ventral insular cortices were also found in the left pars opercularis (Mann-Whitney U = 328.0, p = 0.03622, disgust mean = 0.0469 [SD = 0.0282]/control mean = 0.0603 [SD = 0.0336]), right posterior cingulate (Mann-Whitney U = 333.0, p = 0.04250, disgust mean = 0.0673 [SD = 0.0525]/control mean = 0.0518 [SD = 0.0535]), and the left bank of the superior temporal sulcus (Mann-Whitney U = 314.0, p = 0.02257, disgust mean = 0.0601 [SD = 0.0648]/control mean = 0.0630 [SD = 0.0286]).

## Discussion

The current study revealed that listening to sounds of disgust induces changes in HEFs. Behavioral data indicated that the sound stimuli affected the subjective emotional state by increasing disgust emotion as well as decreasing happiness. As the source current estimation by subtracting disgust minus control conditions revealed the activation, not the suppression, of bilateral insular cortices and inferior frontal cortices, the main effect provoked by the current stimuli was associated with the evoked disgust emotions than the suppressed happiness. Since several studies have reported there to be a relationship between disgust and insular cortical activity (65, 66), the most prominent locus affecting the alteration of HEFs was insular activities evoked by the subjective experience of disgust. In addition, the physiological result on the heart rate variability could not demonstrate the statistical differences in the peripheral cardiac effects such as heart rate, SDNN, and RMSSD on the contrary to our hypothesis. Since our study did not utilize the other indices, such as gastrointestinal information, it would be difficult to further evaluate the initial peripheral effect by means of these physiological data alone. Taken together, the psychophysiological results indicated the alteration of HEFs found in the current study were affected by subjective feeling of disgust, without significant changes in tonic myocardial activities detected by the heart rate variability analysis.

Besides the neural transfer relating to the myocardial activities, there assumed to be alternative conduit such as the humoral pathways involving circulating cytokines, and direct involvement *via* immune cells from the periphery. In addition, interoceptive processing is modulated by respiratory (67), gastrointestinal (68), and genito-urinary information, in addition to the cardiovascular status. Given the nature of interoception, alternative analysis by measuring spontaneous activity towards other organs may reveal the different characteristics of interoceptive processing; however, the temporal advantages of utilizing MEG would be the most efficient for analyzing relatively highly frequent and periodic responses, i.e., myocardial activity, based on rather accumulating evidence. Since the current study utilized disgust emotion mainly affecting the insular cortices, the afferent information was delivered by either autonomic or somatosensory pathway. The former was mainly carried *via* the parasympathetic vagal nerve, which projects mainly to the anterior ventral insular cortices, whereas the latter partially projected directly through the posterior insular cortices. In order to investigate the evoked responses as a carrier signal from periphery, and representing cortical/subcortical patterns of activity, we have estimated the source-evoked time courses and ITCs by dividing the insular cortices in each hemisphere into three sub-regions (anterior dorsal, anterior ventral, and posterior insular cortices). No significant between-condition changes in source-evoked time courses were found, however, the significant differences in ITC—increasing at 19 and 23 Hz and decreasing at 14 Hz—were found only at the right anterior ventral insular sub-region. In addition, statistical analysis of PLIs using the Mann-Whitney U test revealed a significant connectivity change between the right anterior ventral insular cortex and left VMPFC. Since the current results were calculated by averaging the MEG signals triggered by spontaneously delivered bioelectrical cues directly from living organs (individual heartbeats), not by externally supplied cues, the averaged responses reflect more indigenous and induced activities. The current results support our hypothesis that spontaneous heartbeats evoke cortical activity that is associated with the perceptive consequences of inner bodily states according to emotional states. In particular, when subjects felt disgust, brain activity in the right insular cortices and connectivity of beta frequency between the right anterior ventral insular cortices and VMPFC increased. These results suggest that the insular cortices are core regions that mediate the physiological changes modulated by emotional state, and closely associated with the VMPFC in the processing and propagation of information to other brain regions. Although previous studies (69–71) have suggested that the processing of somatic information was performed with low beta frequency (14 to 24 Hz), our results provide additional information of the frequency property within the low beta frequency range both for suppression of 14 Hz and amplification of 19 and 23 Hz when people experience emotions as a consequence of their inner bodily state.

Beyond cortico-cortical networks in the brain, Harrison et al. (68) have reported there to be a relationship between cerebral blood flow and peripheral bodily status. The authors evaluated this correlation using both cardiac and gastric response, which were measured using a combination of functional magnetic resonance imaging (fMRI) and simultaneous recording of multiorgan physiological responses while observing motion pictures of disgust. The authors used two distinct forms of disgust that are defined using functionally different domains (72), i.e., “core disgust” and “body-boundary-violation (BBV) disgust” (73), which elicited two independent patterns of peripheral physiological responses as well as cortical activation. “Core disgust” refers to the feeling related to protecting the body from disease or infection (74–77), is usually provoked by images of the ingestion of spoiled food or vomit, and elicits changes in gastric and right dorsal insular activities. In contrast, “BBV disgust” is related to the denial of mortality, is usually induced by images of body mutilation, blood, and venesection, and evokes changes in cardiac power and left dorsal anterior insular cortex activity. Indeed, “core disgust” induces feelings of nausea, retching, and rapid-gastric activities (78–80), whereas “BBV disgust” evokes feelings of dizziness and lightheadedness, affects cardiovascular responses, and in extreme forms can manifest as vasovagal syncope for 3%–4% of the population (81). Interestingly, both types of disgust stimuli have been found to evoke similar activity in the anterior insula; “core disgust” elicited more activity in the ventral mid-anterior region, while “BBV disgust” activated the primary sensorimotor cortices and superior parietal region (82). Thus, the insular cortices may be the regions in which emotions are embodied, whereby inner bodily states are projected to the brain to perceive and regulate the subjective experience of emotions. Interoceptive afferent signals are conveyed to the dorsal posterior insular cortices, where a highly resolved and somatotopically organized cortical representation of the physiological condition of all bodily tissues is provided (83). Emotionally salient information is replicated repeatedly along regions spanning the anterior to posterior insular cortices, which enables progressive incorporation with multimodal information. The anterior insular cortices are connected to the amygdala, cingulate, ventral striatum, prefrontal, and multimodal sensory regions (84). These connections integrate homeostatic conditions (85) according to motivational, hedonic, cognitive, and social signals, which ultimately form the corresponding emotional state. Our results support these concepts and suggest that listening to sounds of disgust affects activity in the right anterior insular cortices, thereby forming the subjective experience of emotion.

The insular cortices have been identified as the main cortical target of the interoceptive information by organizing topographical bodily maps documented in contemporary neuroscience (5, 14, 83, 84, 86). Afferent feedback from bodily states will eventually be represented in the specific brain network that supply neural substrates of visceral feeling and interoceptive status. The VMPFC is also involved in this network as the trigger region to re-activate somatic patterns, and also as the endpoint where bodily changes in response to brain activity are referred back *via* the somatosensory and insular cortices (87). In addition, recent functional neuroimaging studies have also implicated the insular cortices in both interoceptive processing and emotional feelings (8, 9, 66) in collaborating with somatosensory cortices, such as SI and SII (29, 33), as well as the specific cortical network of default mode (34). Abnormal activity of cortical network implicated in these regions, i.e. high resting-state activity in the insular and limbic/subcortical regions as well as the hypoactivity in higher-level cortical regions, are associated to the clinical conditions of depressive state, including anhedonia, hopelessness, apathy, and particularly dysphoria (88), in extremely forms manifesting, e.g., Cotard syndrome (89). The insular cortices play a key role in executing these functions by representing and projecting information from the sensory feedback of inner bodily status (3, 90).

This study has several limitations that should be mentioned. First, it is unclear whether R or T of ECG triggering were suitable for averaging the HEFs. Although several reports have used T trigger averaging (34, 91), our alternative analysis using T peaks did not clearly identify major components as reported in previous studies. Given the nature of ECG characteristics, the R peak is more prominent than T triggers; averaging using R enables more robust, stable, and technically easier applications without any jittering. Therefore, the HEF components in our study may have been contaminated by ECG T wave components, as the T wave follows the R peak by approximately 400 ms. More convenient and reliable methods for averaging with the T peak should be evaluated. Future studies should examine whether utilizing R or T in individual ECG is superior for obtaining HEFs. The second limitation is related to individual differences in the strength of HEFs. The variations of interoceptive awareness mean that the strength of HEFs thereby vary considerably (18). These variations could be influential distracters in grand-averaging across subjects. Given the premise that one subject may have a greater influence in grand-averaging, an individual with greater responses would more strongly affect the tendency. As our results may contain individual differences, grand-averaged data could reflect unequal contributions among subjects. The third limitation is that our stimuli induce “core” disgust, which mainly results in a feeling of dysphoria in the stomach, without any quantifications of gastrointestinal activity. By excluding BBV disgust, such as bleeding or body mutilation, the stimuli may still have been insufficient for induction of pure “core” disgust due to the limited selection from IADS. As we could not fully evaluate the peripheral effects on interoceptive processing, including cardiovascular as well as gastrointestinal activity, the whole picture of interoceptive carrier and the interpretations on cortical or subcortical reflections still remain uncertain. In fact, gastrointestinal information was also carried regarding the chemical and inflammatory environment as well as the composition of gut bacteria and endocrine responses, is known to modify emotional function (92, 93). Indeed, the responses evoked by the current study may contain all the consequences resulting from presentation of the stimuli, thus future study is required to clarify the characteristics of interoceptive afferent information not only within the regional carrier but also beyond the cortical or subcortical activity constituting brain network, by supporting with the reinforced findings both on behavioral and physiological data; here, the nature of information propagation may help our understanding of further forms of interoception associating to the clinical conditions with aberrant interoceptive processing.

Despite these limitations, the neuromagnetic dynamics demonstrated in the current study revealed HEF-evoked alterations in both activity and connectivity that were affected by emotional experiences. These results provide insights into the frequency properties of information propagation that occurs in the specific beta frequency. This novel method used heartbeat rhythms originating internally, i.e., spontaneous activity of a living organ and not using external stimuli, to determine how indigenous and induced brain activity develop. Moreover, this made it possible to adopt a block design, which is frequently used in fMRI and other brain imaging studies in translational research. Emotional experiences evoked by passive listening to sounds of disgust modulate the autonomic system, which can potentially give rise to alterations in HEFs *via* changes in activity of the right insular cortices and connectivity changes to the VMPFC.

## Conclusion

The alteration of HEFs was investigated in 39 healthy volunteers in association with the subjective feeling of disgust, which evoked right insular cortical activity and the enhancement of cortical connectivity, within the beta frequency range, between the right anterior ventral insular cortex and the left ventromedial prefrontal cortex. Furthermore, the inter-trial coherence in the right anterior insular cortices increased at 19 Hz and 23 Hz, and decreased at 14 Hz, which highlights the involvement of low beta oscillations in emotional processing. The corticolimbic network, particularly involving the insular cortices, plays an important role in emotional regulation and perception. The current results provide a novel insight into the frequency properties of emotional processing, and suggest that emotional arousal impacts the autonomic nervous system, thus giving rise to the alteration of HEFs *via* connectivity changes in the anterior ventral insular cortex and ventromedial prefrontal cortex.

## Data Availability Statement

The raw data supporting the conclusions of this article will be made available by the authors, without undue reservation.

## Ethics Statement

The studies involving human participants were reviewed and approved by Ethics Committee of Gunma University Graduate School of Medicine. The patients/participants provided their written informed consent to participate in this study.

## Author Contributions

YK and YT conceptualized, designed, and conducted the study. SU supported data analysis and interpretation. MM and MF supported the interpretations. YK and all co-authors corrected the manuscript. All authors contributed to the article and approved the submitted version.

## Funding

This work was supported by the Japan Society for the Promotion of Science (Grant-in-Aid for Scientific Research on Innovative Areas; Science of personalized value development through adolescence: integration of brain, real-world, and life-course approaches), area B01 (16H06397), and Grants-in-Aid for Scientific Research B (15H04890, 16K19748). This work was also partially supported by the Japan Agency for Medical Research and Development, AMED (Strategic Research Program for Brain Sciences, Integrative Research on Depression, Dementia and Development Disorders [IR3D]; Development of diagnostic and therapeutic methods for depressive symptoms based on mesocorticolimbic system).

## Conflict of Interest

The authors declare that the research was conducted in the absence of any commercial or financial relationships that could be construed as a potential conflict of interest.
