# Supplementary material for: Alterations of Heartbeat Evoked Magnetic Fields Induced by Sounds of Disgust
Source: Front Psychiatry. 2020 Jul 22;11:683. doi: 10.3389/fpsyt.2020.00683 (PMC7387694; doi:10.3389/fpsyt.2020.00683)
Supplement: Supplementary file 1 [file Table_1.docx]

Supplemental Table 1: Individual result of Heart Rate, standard deviation of all NN intervals (SDNN), and root mean square of successive differences of successive NN intervals (RMSSD)
